# Prostaglandin E Receptor 4 Antagonist in Cancer Immunotherapy: Mechanisms of Action

**DOI:** 10.3389/fimmu.2020.00324

**Published:** 2020-03-10

**Authors:** Yukinori Take, Shinichi Koizumi, Atsushi Nagahisa

**Affiliations:** ^1^AskAt Inc., Nagoya, Japan; ^2^DNA Partners, Fujisawa, Japan

**Keywords:** EP4 antagonist, PGE_2_, NK-DC crosstalk, cancer-immunity cycle, cancer immunotherapy, immune checkpoint inhibitor (ICI), inflamed tumor, non-inflamed tumor

## Abstract

A highly expressed prostaglandin E_2_ (PGE_2_) in tumor tissues suppresses antitumor immunity in the tumor microenvironment (TME) and causes tumor immune evasion leading to disease progression. In animal studies, selective inhibition of the prostaglandin E receptor 4 (EP4), one of four PGE_2_ receptors, suppresses tumor growth, restoring the tumor immune response toward an antitumorigenic condition. This review summarizes PGE_2_/EP4 signal inhibition in relation to the cancer-immunity cycle (C-IC), which describes fundamental tumor-immune interactions in cancer immunotherapy. PGE_2_ is suggested to slow down C-IC by inhibiting natural killer cell functions, suppressing the supply of conventional dendritic cell precursors to the TME. This is critical for the tumor-associated antigen priming of CD8^+^ T cells and their translocation to the tumor tissue from the tumor-draining lymph node. Furthermore, PGE_2_ activates several key immune-suppressive cells present in tumors and counteracts tumoricidal properties of the effector CD8^+^ T cells. These effects of PGE_2_ drive the tumors to non-T-cell-inflamed tumors and cause refractory conditions to cancer immunotherapies, e.g., immune checkpoint inhibitor (ICI) treatment. EP4 antagonist therapy is suggested to inhibit the immune-suppressive and tumorigenic roles of PGE_2_ in tumors, and it may sensitize the therapeutic effects of ICIs in patients with non-inflamed and C-IC-deficient tumors. This review provides insight into the mechanism of action of EP4 antagonists in cancer immunotherapy and suggests a C-IC modulating opportunity for EP4 antagonist therapy in combination with ICIs and/or other cancer therapies.

## Introduction

Increased expressions of PGE_2_ and cyclooxygenase 2 (COX-2), a key enzyme for PGE_2_ synthesis, are routinely identified in a variety of tumor tissues in human and animals, and the contributions of PGE_2_ in tumor initiation, proliferation, and metastasis have been reported (1, 2). Inhibition of the PGE_2_ signal by non-steroidal anti-inflammatory drugs (NSAIDs) or COX-2 inhibitors has been shown to suppress tumor growth in animal tumor models (1, 3). However, cardiovascular and gastrointestinal safety concerns at high doses may have prevented further development of the drugs in human. To avoid the toxicity and achieve intrinsic efficacy of this mechanism, discovery of drugs that inhibit the downstream signaling of PGE_2_ has continued.

Tumors directly or indirectly upregulate the expression of PGE_2_ in tissues, and the highly expressed PGE_2_ regulates tumors and other cells present in the tumor microenvironment (TME), leading to tumor growth. PGE_2_ receptor EP4 is a Gs protein-coupled receptor and activates cAMP-ERK and PI3K signaling (4). EP4 receptors are expressed on the surface of tumor cells, fibroblasts, and immune cells in tumor stroma (2). Recent research evidence suggests that the effects of PGE_2_ on immune cells in the TME actively trigger tumor immune evasion and influence tumor cell growth and patient survival (5). Furthermore, animal experiments using tumor models suggest the involvement of EP4 receptor on immune cells in tumor growth (6–10). The mechanism of action of EP4 signal inhibition in cancer immunotherapy, however, has not yet been clearly demonstrated.

Advances in a therapy using immune checkpoint inhibitors (ICIs) have led to remarkable outcomes in several solid tumors and have established principles of cancer immunity in clinical fields; however, there are still significant limitations with the ICI therapy. The cancer-immunity cycle (C-IC) concept has clearly demonstrated the mechanisms of immune cells to attack and kill tumors through CD8^+^ T-cell cytotoxicity (11) and represented a basis for understanding the present state of cancer immunotherapy, contributing to drug selection and combination therapies for patients refractory to ICIs and conventional therapies (12, 13). This review summarizes the roles of PGE_2_ and EP4 signaling in the concept of C-IC and proposes a position and opportunities for EP4 antagonist therapy in cancer immunotherapy, including in combination with ICIs and other therapies.

## Suppression of Antitumor Immunity by PGE_2_-EP4 Signal

### Inhibition of Conventional Dendritic Cell (cDC) Recruitment Into Tumor by PGE_2_-EP4 Signaling

#### PGE_2_ Inhibits cDC Recruitment Into Tumor Through a Suppression of NK-DC Crosstalk

**Figure 1:**
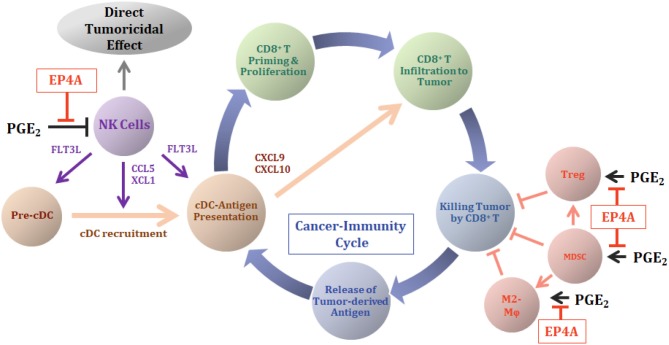
Antitumor mechanism of EP4 antagonist in cancer-immunity cycle (C-IC). The role of the highly expressed PGE_2_ in tumors and antitumor mechanisms of action of EP4 antagonist (EP4A) therapy are illustrated with the concept of the C-IC. The C-IC includes cyclic steps: a release of tumor-derived antigens (tdAs) to the TME, an antigen presentation of cDCs and a priming of CD8^+^ T cells and proliferation in tumor-draining lymph node, translocation of effector CD8^+^ T cells into the tumors, and killing of the tumor cells by the effector CD8^+^ T cells, and the release of tdAs again. Tumor cell death results in a release of damage-associated molecular patterns (DAMPs), which contains molecules that can be recognized as antigens to specify the tumor cells in the body. Conventional dendritic cells (cDCs) capture and process these tdAs and prime the naïve CD8^+^ T cells. During the ICI therapy, continuous supply of the pre-cDCs from bone marrow is required for the consecutive continuation of the C-IC result in the success of ICI therapy. NK cells in tumors secrete XCL1 and CCL5 that induce an infiltration of cDCs into the TME. The NK cells further secrete a growth factor FLT3L, which supports survival of cDCs and enhances local cDC differentiation. In TME with high levels of PGE_2_, the PGE_2_ shut off supply of cDCs into the TME through the inhibition of NK cell functions, thereby causing disruption of the C-IC and the failure of ICI therapy. The cDCs located in the tumor further secrete chemokines CXCL9/10 that attract CD8^+^ effector T cells into the tumor. EP4A therapy inhibits the effects of PGE_2_ on the NK cells and revitalizes the supply of cDCs into the TME. Highly expressed PGE_2_ in tumors increases populations of immune-suppressive cell species, MDSC, Treg, and M2-like macrophage (M2-Mϕ) in the TME and inhibits cytotoxic ability of effector CD8^+^ T cells against tumor cells. The tumor-produced PGE_2_ regulates these immune cells directly or indirectly and produces the non-T-cell-inflamed tumor environment, accelerating tumor immune evasion. EP4A inhibits tumor-mediated PGE_2_ functions on the immune-suppressive cell species and eliminates the elements causing disruption of an effective C-IC in cancer immunotherapy.

Clinical evidence demonstrated that patients with a relatively high number of immature DCs in the tumor had longer overall survival. A decrease in the number of DCs in the tumor significantly correlated with advanced tumor stage in patients with colorectal cancer (14). Recent research results suggest that a conventional DC1 (cDC1, CD103^+^ DC in mice and CD141^+^/BDCA3^+^ DC in human) has been reported as the only professional antigen-presenting DC that can capture tumor-specific antigen on their MHC class I molecule and cross-present the antigen to CD8^+^ T cells (15–17). The recruitment of cDC1 into the TME is critically supported by natural killer (NK) cells in the tumors by secreting cDC1 chemoattractants XCL1 and CCL5. In patients with cancer, intratumoral XCL1 and CCL5 levels correlate with the presence of NK and cDC1 cells in the tumor and are associated with increased overall survival (18). NK cells also produce formative cytokine for cDC1, Fms-related tyrosine kinase 3 ligand (FLT3L), and stably form conjugates with cDC1. The NK-DC crosstalk positively regulates cDC1 abundance in melanoma. The FLT3L further leads to the expansion of cDC progenitors in bone marrow and promotes the accumulation of immature cDC1 at the tumor site (19). In human melanoma, the numbers of NK cells and cDC1 are demonstrated to correlate with increased overall survival and patient responsiveness to anti-PD-1 immunotherapy (20, 21). Importantly, the tumor-produced PGE_2_ impairs the viability and chemokine-producing property of NK cells and downregulates chemokine receptor expression in cDC1s in the TME, resulting in cancer immune evasion (18). Thus, the inhibition of NK cell functions by PGE_2_ and subsequent suppression of the NK-DC axis is suggested as the key trigger to induce the absence and functional failure of cDCs in the TME and the disruption of C-IC (Figure 1).

#### EP4 Signal Inhibits NK Cell Functions Following cDC Recruitment Into TME

NK cells have direct cytotoxicity especially to MHC class I molecule-downregulated tumor cells. EP4 signal inhibition is reported to decrease the expression of the MHC class I on mammary tumor cells and stimulates antitumor effects of NK cells (22). PGE_2_ secreted by thyroid cancer cells promote antitumor immune suppression through the inhibition of NK cell cytotoxicity via EP2 and EP4 receptors (23). EP4 signaling, in addition to the direct antitumor cytotoxicity of NK cells, has demonstrated tumoricidal efficacy through diverse interactions within the immune system. A direct treatment of PGE_2_ or EP4 agonist to murine splenic NK cells *in vitro* blocked NK cell functions such as IFNγ and TNFα productions and cancer cell migration (24). The re-activation of NK cell functions in tumor-bearing mice was reported via an *ex vivo* study using the EP4 antagonist RQ-15986. RQ-15986 was administrated systemically to 66.1 breast tumor-bearing mice for 3 weeks and then splenic NK cells were isolated and tested for their ability to produce IFNγ by IL-2 stimulation. Although NK cells isolated from tumor-bearing mice lost their ability to produce IFNγ almost completely compared with that of normal mice, NK cells from RQ-15986-treated tumor-bearing mice completely recovered IFNγ-producing ability (25). Moreover, in an obesity-associated hepatocellular carcinoma mice model, the daily systemic therapy of the EP4 antagonist AAT-008 for 3 weeks showed significant induction of cDC1 (CD103^+^ DC) frequency with no change in the frequency of other DC classes (10). Although no evidence of EP4 receptor inhibition on the NK cells is evaluated in this study, the NK-DC crosstalk-mediated mechanism should be included in the evidence observed as changes on cDCs. Taken together, PGE_2_ is suggested to inhibit cDC1 recruitment into the TME through the suppression of NK cells and the NK-DC crosstalk. The EP4 antagonist restores the cDC1 population in the TME by re-activating NK cells, and then the NK-DC crosstalk, which is suppressed by tumor-induced PGE_2_ (Figure 1).

### cDCs in Tumor Accelerate Effector CD8^+^ T-Cell Infiltration Into the Tumors Through CXCL9/10 Production

The absence of the effector CD8^+^ T cells in tumors correlates with a poor prognosis for clinical outcome in both human and animal cancer models (26, 27). The exclusion of CD8^+^ T cells from the tumor tissue is often observed in many cancers, and they are commonly referred to as non-T-cell-inflamed tumors (28). The non-T-cell-inflamed tumors are difficult to treat with ICIs compared with inflamed tumor therapies in the clinic. A failure in effector CD8^+^ T cells' traffic into melanoma tissue from tumor-draining lymph nodes in a mice model is mediated by the lack of chemokines CXCL9 and CXCL10 produced by CD103^+^ DCs. These DCs are generally present in inflamed tumors, whereas these are absent in non-inflamed tumors (29). Thus, the importance of cDCs in tumors is suggested not only for tumor-specific effector CD8^+^ T-cell production and proliferation but also for the active infiltration of the effector CD8^+^ T cells into the tumor bed. Production of chemokine CXCL10 in human immature DCs is reported to be negatively regulated by PGE_2_ and EP2 and/or EP4 receptors are involved in this effect (30). Treatment with EP4 antagonist E7046 demonstrated a significant increase in CD8^+^ T-cell frequency in tumors in a CT26 colon cancer–bearing mice model (9). The evidence strongly suggests that EP4 antagonist therapy increases the infiltration of effector CD8^+^ T cells into the tumor through the active production of chemokines CXCL9 and 10 from the DCs in tumors corresponding to the restoration of NK cell functions by EP4 signal inhibition (Figure 1).

### PGE_2_-EP4 Signal Activates Suppressive Immune Cells and Inhibits Effector CD8^+^ T Cells

Myeloid-derived suppressor cells (MDSCs), M2-like macrophages in tumors often called tumor-associated macrophages, and regulatory T cells (Tregs) are the major immune-suppressive cell species in the TME. These cells directly or indirectly inhibit cytotoxic activity of effector CD8^+^ T cells against tumor cells, primarily through the production of immune-suppressive cytokines such as IL-10 and TGFβ. The PGE_2_-EP4 signal is demonstrated to activate the functions of these immune-suppressive cells and to promote inactivation of antitumor activity in effector CD8^+^ T cells, leading to tumor immune evasion (Figure 1).

#### PGE_2_-EP4 Signal Regulates MDSC Differentiation and Macrophage Polarization

The MDSCs frequently exist in TME and mediate immune suppression through the production of arginase, inducible NOS, TGFβ, IL-10, and COX-2. These molecules directly or indirectly induce Treg activities (31), suppress NK cell functions (32), and promote the polarization of macrophages toward M2-like properties. They cooperatively and synergistically impair the tumoricidal property of effector CD8^+^ T cells, thereby leading to the evasion of tumor cells from the host's antitumor immunity (33). PGE_2_ is demonstrated to stimulate MDSC differentiation and proliferation in tumors (32, 34–36), and the EP4 and/or EP2 receptors promote the activation. Albu et al. demonstrated the significant increase in the number of MDSCs in CT-26 colon cancer–bearing mice spleen and the almost complete reversal of the number of MDSCs as a result of continuous treatment with the EP4 receptor antagonist E7046 (9).

Macrophages polarize to functionally different M1- and M2-like phenotypes by various stimulation in tissues with inflammation and tumor. In *in vitro* experiments, a human peripheral blood mononuclear cell primary culture in the presence of GM-CSF plus IL-4 promotes differentiation to DCs. An addition of PGE_2_ in this culture suppresses the formation of DCs and skewed the differentiation into the M2-like macrophage. EP4 antagonist E7046 treatment in this culture dose-dependently reversed the effects of PGE_2_ on the proportion of differentiation to DCs or M2-like macrophages (9). Similarly, in human cervical cancer cell culture, PGE_2_ hampered monocyte to DC differentiation and skewed their differentiation toward M2-like macrophages. A depletion of PGE_2_ restored the normal monocyte to DC differentiation (37). In an Apc^min/+^ mice, a deletion of the myeloid EP4 receptor led to the loss of the arginase 1-expressing M2 phenotype macrophage population in a histochemical study (38). In K19-Wnt1/C2mE mice, a PGE_2_ highly producing transgenic gastric cancer mice model, EP4 antagonist RQ-15986 therapy achieved almost complete shrinkage of the tumor. M2-like macrophage polarization was observed in the tumor, and the EP4 signal inhibition by RQ-15986 released this polarization (6). Collectively, the evidence strongly supports the EP4 antagonist's role in the prevention of M2-like macrophage polarization and accumulation in tumors derived by tumor-associated PGE_2_.

#### PGE_2_-EP4 Signal Activates Treg Cells

The frequency of circulating or tumor-infiltrating Tregs is associated with poor survival of patients in many cancers, including colon, breast, melanoma, and lung (39). PGE_2_ is reported to increase the expression of the Treg-specific marker Foxp3 and to stimulate the functions of Treg cells *in vitro* and in a mice lung cancer model. The activated Treg cells also induce COX-2 expression and PGE_2_ production, which then support immune-suppressive functions by themselves. These autocrine and paracrine effects of PGE_2_ cooperatively activate Treg cells in tumors (40, 41). In Treg cell culture isolated from the spleen of EP2 or EP4 knockout mice, expressions of Foxp3 in the presence of PGE_2_ were significantly reduced compared with those Treg cells isolated from normal mice. The contribution of EP2 and EP4 receptors in PGE_2_ signaling in activated Tregs has also been demonstrated using the antagonists AH6809 and AH23848, respectively, by evaluating the reversal of Treg cell-mediated T-cell effector function failure (41). An immunosuppression caused by UV irradiation was mediated through the induction of Treg proliferation, and PGE_2_-EP4 signaling was also reported to be involved (42).

#### Activation of Immune-Suppressive Mechanisms by PGE_2_-EP4 Signal Impairs Effector CD8^+^ T-Cell Functions

The effector CD8^+^ T cells infiltrated to tumors are primarily able to fight against the antigen-targeted tumor cells. However, the tumors activate the previously described immune-suppressive cell species to avoid the attack from effector CD8^+^ T cells. PGE_2_ is one of the key molecules triggering the immune suppression in TME (34). The EP4 antagonist E7046 therapy increased the population of intratumoral effector CD8^+^ T cells, inhibited myeloid MDSC activation, and suppressed the polarization of macrophages to M2-like properties in a CT26 mouse colon cancer model. It showed potent antitumor efficacy compared with non-treated controls (9). The aforementioned evidence strongly suggests that tumor-produced, PGE_2_-activated immune-suppressive cell species in the TME inhibit the tumoricidal function of effector CD8^+^ T cells in the tumor through EP4 signaling, and that the EP4 antagonist rescues the tumoricidal function of CD8^+^ T cells (Figure 1).

## Mechanism of Action of EP4 Antagonist in the C-IC

### Cancer-Immunity Cycle

The concept of the C-IC is proposed by Chen and Mellman and suggests the respective roles of tumor cells, tumor-derived antigens, DCs, and effector CD8^+^ T cells in a series of stepwise events, which proceed and expand iteratively in cancer immunotherapy (11). For patients with cancer with deficiency in immunological antitumor ability, rescuing single or few defects among these steps is essential for the C-IC to optimally perform. Amplifying the entire C-IC will provide effective antitumor activity; however, drugs such as ICI generally target only a single step in the cycle. Therefore, the most effective approaches will require targeting the rate-limiting steps in a given patient using the appropriate combination of drugs (12). The EP4 antagonist is one of the drug classes that can reactivate antitumor immunity, rescuing the defects of the steps in the C-IC as described above. Understanding the mechanism of action of the EP4 antagonist in cancer immunotherapy in combination with the C-IC concept would be a useful procedure when considering therapeutic strategy, drug regimen combination, or radiotherapy in the clinical environment.

### Roles of PGE_2_ and EP4 in C-IC and Perspectives on EP4 Antagonist in Immunotherapy

As summarized in the previous sections, PGE_2_ mediates the immunologically deficient tumor environment by regulating several immune cell species that express the EP4 receptor and develop multiple dysfunctions in the C-IC. The EP4 antagonist is suggested to demonstrate antitumor efficacy by restoring the PGE_2_-mediated dysfunctions in the host's antitumor immune systems. Figure 1 illustrates the roles of PGE_2_ and the EP4 antagonist in cancer immunotherapy combined with the C-IC concept. Tumor-produced PGE_2_ inhibits the NK cell function in the TME and suppresses XCL1 and CCL5 production, which promotes the infiltration of cDC precursors (pre-cDCs) into the TME. The suppression of NK cells further mediates the reduction of cDC function in tumors, with FLT3L playing a key role. Moreover, the decrease in the cDC population and function in the TME causes the reduction of CXCL9/10 production by cDCs and suppresses the translocation of effector CD8^+^ T cells into the tumor from draining lymph nodes. Thus, the PGE_2_-mediated exhausting of NK cell triggers the failure of sequential recruitment of cDCs and the effector CD8^+^ T cells, essential elements of the C-IC, into the TME (43). The EP4 antagonist therapy is demonstrated to block the PGE_2_-mediated dysfunction of NK cells, which enables the continuous infiltration of cDCs into the TME, thereby increasing the population of tumor-specific effector CD8^+^ T cells in the TME (9, 25). The PGE_2_ further activates the functions of immune-suppressive cell species, such as MDSC, Tregs, and tumor-associated M2-like macrophages, thereby directly or indirectly wresting the functions of effector CD8^+^ T cells from the tumor cells. The EP4 antagonist supports the roles of antitumor immune functions by inhibiting the roles of PGE_2_ in the immune-suppressive cells as noted previously (Figure 1).

**Figure 2:**
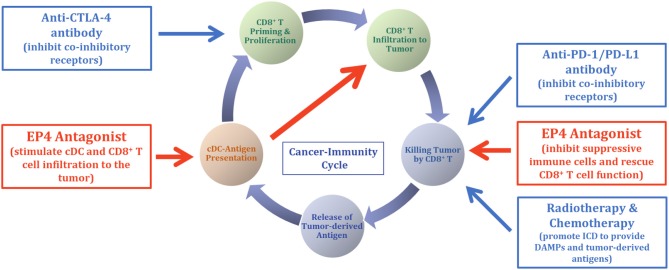
Opportunity for EP4 antagonist therapy in cancer immunotherapy. Based on the mechanisms of action of the EP4 antagonist (Figure 1), opportunities for the EP4 antagonist therapy in cancer immunotherapy are illustrated. Because EP4 antagonist therapy is suggested to change the tumor environment from non-T-cell inflamed to inflamed, concomitant use of EP4 antagonist with ICI will sensitize the efficacy of ICI in some portion of patients whose prior ICI monotherapy was non-responsive. Combination of chemotherapy and radiotherapy with ICI therapy is suggested to show cooperative antitumor efficacy via the production of DAMPs and activation and restoration of tumor-targeted immune responses. This combination is reasonable from the standpoint of C-IC theory, and the addition of EP4 antagonist therapy with ICI, together with chemotherapy and/or radiotherapy, may further accelerate the potential of the C-IC and antitumor therapeutic efficacy.

Therapy using ICIs in humans led to remarkable antitumor efficacy in several tumors, enabling complete elimination of tumors in some cases. However, the proportion of patients who achieve a response remains generally modest. Absence or functional failure of effector CD8^+^ T cells in tumors, the non-inflamed tumor, is suggested to be one reason for being refractory to ICI therapy according to current clinical experience. For a portion of ICI-refractory patients, the failure of C-IC in their TME is suggested. As summarized in this review, tumor-mediated PGE_2_ is one of the key molecules that disrupt the C-IC and promote the non-inflamed tumor environment, resulting in ICI therapy deficiency. When drugs are used concomitantly, EP4 antagonist therapy may sensitize the efficacy of ICI therapy in patients with ICI-refractory by restoring the sequential NK and cDC cell functions. Animal experiments support the combination of the EP4 antagonist E7046 (ER0886046) with anti-CTLA4 in a mouse melanoma B16F10 model and with anti-PD-1 or anti-PD-L1 antibodies in a mouse colon cancer CT26 model. Higher antitumor efficacies were demonstrated in combination, compared with E7046 or the ICI antibody alone (44). Figure 2 illustrates the opportunity to introduce EP4 antagonist therapy with ICIs, which may cooperatively interact leading to an efficient C-IC. An additional opportunity for EP4 antagonist therapy is in combination with the other cancer therapies that induce immunogenic cell death (ICD), such as tumor radiotherapy and some types of cancer chemotherapies. The ICD, by releasing damage-associated molecular patterns (DAMPs) and tumor-derived antigens, has been shown to support the activation of DCs' functions and their traffic to lymph nodes, activating tumor-targeted immune responses (45). The EP4 antagonist E7046 was demonstrated to show improved antitumor efficacy and prolonged survival of mice in combination with radiotherapy, compared with radiotherapy or E7046 alone in a CT26 colon cancer mouse model (46). The combination of radiotherapy and/or chemotherapy with the EP4 antagonist is thought to synergistically accelerate the C-IC. Simultaneous increases in the cDC population by EP4 therapy and the production of DAMPs through therapy-producing ICD in tumors are essentially required for the effective production of tumor-specific effector CD8^+^ T cells (44) (Figure 2).

## Conclusion

Several companies are currently conducting clinical trials of EP4 receptor-selective antagonists for cancer therapy (ClinicalTrials ID: NCT03658772, NCT03696212, NCT03152370, NCT03661632) and evaluating EP4 antagonist therapy in anti-PD-1-refractory tumors, microsatellite stable tumors, and in combination with tumor radiotherapy. Enhanced understanding of the mechanism of action of the EP4 antagonist therapy within the C-IC concept enabled us to pursue a new therapeutic approach to EP4 antagonist therapy.

## Author Contributions

YT wrote the manuscript. SK and AN contributed to the scientific discussion.

### Conflict of Interest

The authors are employees and shareholders of AskAt Inc. AN is also an employee of DNA Partners.
